# The Impact of Deep Brain Stimulation of the Subthalamic Nucleus on Sleep–Wake Function and Circadian Rhythms in Patients with Parkinson's Disease

**DOI:** 10.1002/mdc3.70160

**Published:** 2025-06-04

**Authors:** Julia Steinhardt, Laura Lokowandt, Cosima Xenia Schmidt, Dirk Rasche, Henrik Oster, Britta Wilms, Norbert Brüggemann

**Affiliations:** ^1^ Department of Neurology University of Lübeck Lübeck Germany; ^2^ Center of Brain, Behavior and Metabolism University of Lübeck Lübeck Germany; ^3^ Institute of Neurobiology University of Lübeck Lübeck Germany; ^4^ Department of Neurosurgery University of Lübeck Lübeck Germany; ^5^ Medical Clinic 1 University of Lübeck Lübeck Germany; ^6^ German Center for Diabetes Research (DZD) Neuherberg Germany

**Keywords:** circadian rhythm, deep brain stimulation, sleep, subthalamic nucleus, VTA

## Abstract

**Background:**

Deep brain stimulation (DBS) of the subthalamic nucleus (STN) is a well‐established therapy in advanced Parkinson's disease (PD) improving motor and non‐motor signs including sleep. The mechanisms of sleep improvement are insufficiently understood.

**Objectives:**

The objective was to identify stimulation‐dependent effects on the sleep–wake cycle and circadian clock regulation.

**Methods:**

Fourteen PD patients who underwent STN DBS (PD‐DBS) were assessed before as well as 6 and 12 months post‐surgery. As control groups, 18 PD patients under best medical treatment (PD‐CON) and 25 healthy controls (H‐CON) were also enrolled. Wrist actigraphy to measure sleep and sleep questionnaires, such as the PD Sleep Scale (PDSS), were applied and the expression of clock genes in peripheral blood was measured. Electrode placement in the STN was localized, and the local impact of STN DBS was estimated.

**Results:**

STN DBS improved daytime sleepiness 12 months post‐surgery (*P* = 0.006) which was correlated with limbic VTA (r = 0.737, *P* = 0.006). PDSS scores decreased 6 months post‐surgery (*P* = 0.050) and were positively associated with activation of the motor part of the STN (r = 0.582, *P* = 0.037). Time in bed increased after surgery and was also positively correlated with the activation of the motor part of the STN (r = 0.941, *P* = 0.005). There were no changes in clock gene expression between groups and over time (all *P* ≥ 0.323).

**Conclusions:**

Sleep and daytime sleepiness are improved after STN DBS in PD. Different STN sub‐proportions contribute to these improvements. The changes in sleep appear not be driven by changes in clock gene expression.

PD is a disorder with both motor and non‐motor symptoms, including neuropsychiatric symptoms, cognitive decline, autonomic dysfunction, and sleep disorders.^1^ Sleep disorders occur in up to 96% of patients with PD,^2^ including disturbances in the sleep–wake cycle, sleep fragmentation, excessive daytime sleepiness, rapid eye movement (REM) sleep behavior disorder (RBD), and restless legs syndrome (RLS).^1^, ^2^, ^3^, ^4^, ^5^ Sleep disorders impair not only metabolic health,^3^, ^6^ but also directly impact quality of life (QoL), impair mood, and lead to increased morbidity and mortality.^1^, ^4^

Sleep and wakefulness result from central nervous integration of activating and sleep‐inducing signals modulated by circadian signals. A central pacemaker, the suprachiasmatic nucleus (SCN), located at the base of the hypothalamus, generates stable circadian rhythms. Afferents from the retinohypothalamic tract, enable a constant synchronization of the SCN, with regularly recurring environmental factors, such as light. Clock genes regulate their transcription in a 24‐h rhythm in coupled feedback loops. Heterodimers of the transcription factors Circadian Locomoter Output Cycles Protein Kaput (CLOCK) and Basic Helix–Loop–Helix ARNT Like 1 (BMAL1), the positive elements of this feedback loop, stimulate the transcription of genes of negative factors such as Period 1–3 (*PER1‐3*) and Cryptochrome 1/2 (*CRY1*/2). This negative feedback, in turn, inhibits the transcription of *CLOCK* and *BMAL1*, ending in a periodic transcriptional activation and deactivation occurring every 24 h.^7^, ^8^

Over the last 30 years, deep brain stimulation (DBS) of the subthalamic nucleus (STN) has become a neurosurgical standard therapy for treating therapy‐resistant tremor or motor complications in advanced PD stages.^9^, ^10^, ^11^ Growing evidence indicates that alterations in the circadian system might contribute to sleep–wake cycle dysregulation and other non‐motor symptoms in patients with PD.^3^ DBS has a positive impact on different aspects of sleep, such as improved sleep quality and several sleep parameters, such as total sleep time, wakefulness after sleep onset, and sleep efficiency.^12^ It is still unclear how STN DBS might affect circadian clock gene expression in connection with the exact electrode position in a longitudinal design.

The present study aims to investigate the impact of STN DBS on sleep patterns and clock gene expression in patients with PD longitudinally with a specific focus on the contribution of different subparts of the STN. Emphasis was placed on changes in the sleep–wake cycle, sleep architecture, and clock rhythms in response to the degree of motor, limbic, or associative STN DBS modulation. The following hypotheses were tested: (i) PD patients with STN DBS will show improved sleep quality compared to pre‐surgery states. (ii) Changes in clock gene expression are observed in PD patients with STN DBS compared to control subjects. (iii) The electrode position within the STN concerning the sub‐territories correlates with changes in sleep parameters.

## Methods

Details of the study were published previously.^13^ Briefly, the study included 14 PD patients who underwent STN DBS (PD‐DBS), 18 PD patients with the best medical treatment as the disease control group (PD‐CON), and 25 healthy control subjects (H‐CON). All patients fulfilled the diagnostic criteria of the Movement Disorder Society.^14^ Groups were matched for age, gender, body mass, and BMI, while both PD groups were also matched for age of disease onset and disease duration. PD‐CON group fulfilled the criteria for DBS surgery but refused to perform the surgery. All patients continued to take their dopaminergic medication throughout the study (Med ON). Cognitive function was tested using the Montreal Cognitive Assessment (MoCA).^15^, ^16^ Depressive symptoms were assessed by the Beck Depression Inventory (BDI2). Before participation, informed written consent was obtained from all participants. The study was approved by the local ethics committee of the University of Lübeck (AZ17‐198) and conducted according to the ethical standards of the Declaration of Helsinki.

## Study Design and Measurement of Sleep

The participants were studied at four different time points within 12 months of the study duration. T_0_ was the baseline measurement ~2 weeks before DBS surgery (PD‐DBS). The assessment was usually performed before potential preoperative adjustments of the PD medication. The second time point of measurement (T_3M_) was 12 weeks after DBS surgery (PD‐DBS) or after T_0_ (PD‐CON, H‐CON). The third time point (T_6M_) was 24 weeks after DBS surgery (PD‐DBS) or after T_0_ (PD‐CON, H‐CON), and the last time point (T_12M_) 52 weeks after DBS surgery (PD‐DBS) or after T_0_ (PD‐CON, H‐CON), respectively.

Sleep features were assessed using the Motionwatch 8 actigraphy system (MW8, CamNtech; Cambridge, UK) for seven consecutive days on their non‐dominant wrist.^17^, ^18^ Participants were instructed to press the event marker button each night when they went to bed and started trying to sleep and again each morning when they woke up. For the analysis, the mean of six consecutive days, including five working days and one day of the weekend, were used, since 75% did wear the MW8 one day less than specified. Data were analyzed using MotionWare 1.0.27 software (CamNtech, Cambridge, UK), and the following sleep indices were calculated: actual sleep time (total time spent sleeping), time in bed, and sleep efficiency.

In addition, participants completed the Epworth Sleepiness Scale (ESS)^19^ and the Parkinson's Disease Sleep Scale (PDSS).^2^ The ESS was used to assess daytime sleepiness and scores > 10 (of 24) are considered as excessive daytime sleepiness and scores > 15 (of 24) as severe sleepiness. The PDSS assessed nocturnal sleep disturbance scored from 0 to 60.

### Clinical and Metabolic Assessments

Participants were neurologically examined in Medication ON and DBS‐ON states according to the Unified Parkinson's Disease Rating Scale (MDS‐UPDRS‐III) and Hoehn and Yahr Scale.^14^ The levodopa‐equivalent doses (LED) were calculated to estimate the equivalent amount of the total individual anti‐parkinsonian drugs.^20^ We also assessed other medications that could potentially affect sleep (sleeping aid, antidepressive medication). The frequency of such medication, however, was overall low in both PD groups (T_0_ N = 0/1, T_6M_ = 2/1, T_12M_ = 3/1).

Blood samples for the analysis of clock genes were taken under fasting conditions at the time points T_0_, T_6M_, and T_12M_ at 9 A.M. Samples were centrifuged, aliquoted, and then frozen at −80°C until analysis. For the analysis of the cell blood count, samples were prepared for analysis in the LADR Laboratory (Geesthacht, Germany). The Paxgene samples were taken and prepared according to the manufacturer's instructions and then frozen at −80°C until analysis.

### Clock Gene Analysis

RNA was isolated from whole‐blood samples using the PAXgene Blood RNA Kit (PreAnalytiX, Hombrechtikon, Switzerland). cDNA was synthesized from RNA using random primers and the High‐Capacity Reverse Transcription Kit (Applied Biosystems, Waltham, USA). cDNA samples were diluted 1:5, and quantitative real‐time PCR (qPCR) was performed using the Go Taq qPCR Master Mix (Promega, Madison, USA). The qPCR reactions were carried out on a CFX96 thermocycler (Bio‐Rad, Hercules, USA). The following primer sequences were used: *EEF1A1* forward 5′‐TGCCCCAGGACACAGAGACTTCA‐3′, *EEF1A1* reverse 5′‐AATTCACCAACACCAGCAGCAA‐3′, *PER3* forward 5′‐TGAAGAATCCATCCCATCCTACTG‐3′, *PER3* reverse 5′‐TATACTGCTGTCGCTGCTTCC‐3′, *CLOCK* forward 5′‐ACCCTTCCTCAACACCAACCA‐3′, *CLOCK* reverse 5′‐ATGCGTGTCCGTTGTTCCAA‐3′, *BMAL1* forward 5′‐GTACCAACATGCAACGCAATG‐3′, *BMAL1* reverse 5′‐TGTGTATGGATTGGTGGCACC‐3′, *NR1D2* forward 5′‐CAGCAATGTCGCTTCAAAAA‐3′ and *NR1D2* reverse 5′‐TGGTCTTCATTGCACTTTGC‐3′. Target gene C_t_ values were normalized to those of *EEF1A1* to calculate relative expression values. As an indicator of clock phase, a clock phase ratio between gene expression levels of the positive and negative arm components of the circadian feedback loop (PER3*NR1D2/BMAL1*CLOCK) was determined.

### Electrode Localization

Lead‐DBS localization was performed with Lead‐DBS. In short, we used the obtained pre‐and postoperative MR images and used the LEAD DBS toolbox version 2.3.1 within MATLAB 2019 (The MathWorks, USA). Images were linearly coregistered to the pre‐operative T1‐image using SPM12. Co‐registration, normalization, and a brain shift correction were performed. Electrode trajectories were then manually adjusted to fit the visible artifacts in the postoperative image optimally. The individual lead reconstruction as well as the stimulation parameters was used to calculate the corresponding approximation of the DBS‐activated tissue in the brains of PD patients.^21^, ^22^ For details, please see Steinhardt et al (2023).^13^

### Statistics

Data are given as mean ± SD or mea ± SEM (figures). Excel Version 2016 (Microsoft, Redmond, WA, USA), Jamovi Version 1.8.4, and GraphPad Prism version 8.0 (La Jolla, CA, USA) were used for analysis. One‐way ANOVA was used to test for baseline differences between the metric data. Variables were checked for normality using the Kolmogorov–Smirnov test. Sphericity was tested using Mauchly's W. In the case of non‐sphericity, Greenhouse–Geisser correction was applied. The analyses of the data over time were based on a mixed general linear model (GLM), including the main factors “Group” (PD‐DBS vs. PD‐CON vs. H‐CON) and “Timepoint” (T_0_, T_6M_, T_12M_). If GLM resulted in a significant F value with *P* ≤ 0.05 for a main effect or interaction, post hoc Student's t‐tests were performed using Bonferroni‐Holm‐correction. A *P*‐value <0.05 (after correction) was considered significant. Pearson's correlation coefficients were used for clinical data, VTA, and primary outcome parameters to test for a significant association between the two parameters. Only significant correlations are reported.

This manuscript is based on data of a subsample of a recent study comparing body mass index and body weight trajectories between PD patients treated with STN DBS compared with PD patients under best medical treatment and healthy controls before and 12 months post‐surgery^13^ with an effect size of d = 2.14. The subjects of the present manuscript are a subsample of this group, where data on sleep and clock gene expression were available. Upon the effect size of the main outcome parameter, a sample size of six subjects per group would have been required. For analysis of the secondary outcome parameters sleep and clock genes a power lowered by 2/3rd would have been assumed, revealing 12 subjects per group (d = 0.70; alpha = 0.05; 1‐ß = 0.80).

## Results

Table 1 shows baseline characteristics. No significant demographic differences existed between all groups at baseline (*P* ≥ 0.118). The stimulation parameters are provided in Table S1 and group‐level electrode localization is shown in the Figure S1.

**TABLE 1:** Demographic and disease‐specific data of participants at T_0_

Baseline (T_0_)	PD‐DBS	PD‐CON	H‐CON	*P*
*N*	14	18	25	
Age (years)	56.6 ± 8.4	57.9 ± 7.9	59.4 ± 8.0	0.590
Body mass (kg)	81.4 ± 17.5	81.8 ± 14.0	77.3 ± 11.7	0.526
BMI (kg/m^2^)	26.7 ± 4.3	26.3 ± 4.2	25.6 ± 3.3	0.682
Gender (male/female)	8/6	13/5	12/13	0.267
MoCA (Score)	25.6 ± 1.7	28.9 ± 1.3	28.0 ± 1.6	0.118
MDS‐UPDRS‐Total ON (Score)	56.5 ± 16.3	46.8 ± 18.4	4.5 ± 3.1	0.124^†^
MDS‐UPDRS‐I ON (Score)	9.6 ± 5.8	10.3 ± 6.1	2.7 ± 2.5	0.948^†^
MDS‐UPDRS‐II ON (Score)	13.4 ± 7.8	9.7 ± 6.2	0.5 ± 0.9	0.140^†^
MDS‐UPDRS‐III ON (Score)	32.0 ± 8.1	25.2 ± 8.4	1.8 ± 1.7	0.021^†^
MDS‐UPDRS‐IV ON (Score)	6.1 ± 4.6	2.1 ± 2.4	n.a.	<0.001^†^
Hoehn & Yahr Stage (ON)	2.0 ± 0.3	2.0 ± 0.5	n.a.	0.622^†^
L‐Dopa equivalent dose (mg/day)	833 ± 491	796 ± 513	n.a.	0.838^†^

*Note*: Results are expressed as mean values±SD. Hoehn & Yahr stage is represented as median values±SD.

^†^ ANOVA revealed the difference between PD‐DBS and PD‐CON.

Abbreviations: H‐CON, healthy control subjects; LED, levodopa equivalent dose; MDS‐UPDRS, Movement Disorder Society—Unified Parkinson's Disease Rating Scale; MoCA, Montreal Cognitive Assessment; PD‐CON, patients with PD under best medical treatment; PD‐DBS, patients with PD that underwent DBS surgery.

### Effects of STN DBS on Motor Symptoms and Medication

As assessed by UPDRS part IV, motor complications related to L‐dopa treatment were improved after surgery in the PD‐DBS group over time, while not in PD‐CON (time × group interaction: *P* ≤ 0.001). For PD‐DBS, motor complications decreased from 6.1 ± 4.6 at T_0_ to 2.8 ± 2.9 at T_6M_ (*P* = 0.009) and remained stable at 2.7 ± 3.1 after 12 months (*P* = 0.900). In PD‐CON, motor complications were less severe than in PD‐DBS and remained unchanged (T_0_: 1.9 ± 2.2; T_6M_: 2.1 ± 1.9; T_12M_ to 3.1 ± 3.0; all *P* ≥ 0.900; see Table 2). As previously reported,^13^ LED decreased over time. This effect was mediated by lower values in PD‐DBS after surgery (time × group interaction (*P* ≥ 0.016; F = 4.48)). LED decreased from 833 ± 491 mg/day at baseline by 42% to 486 ± 406 mg/day at T_6M_ (*P* = 0.010) and remained stable at 428 ± 292 mg/day after 12 months (*P* > 0.900) in PD‐DBS. LED remained stable over time in PD‐CON (T_0_: 764 ± 512 mg/day; T_6M_: 765 ± 475 mg/day; T_12M_: 689 ± 400 mg/day, all *P* ≥ 0.888). The stimulation settings can be found in the supplementary material of Steinhardt et al (2023).^13^

**TABLE 2:** Changes in UPDRS‐III motor scores over time in all groups

Timepoint	PD‐DBS	PD‐CON	H‐CON	*P*‐value
MDS‐UPDRS‐III (T_0_)	32.0 ± 8.1	25.4 ± 8.6^‡^	1.8 ± 1.7^§^, ^¶^	Time: *P* < 0.001 (F = 19.2) Group: *P* < 0.001 (F = 63.8) Time × Group: *P* < 0.001 (F = 8.2)
MDS‐UPDRS‐III (T_6M_)	19.5 ± 7.3^†^	19.6 ± 9.1	1.4 ± 1.6^§^, ^¶^	Time: *P* < 0.001 (F = 19.2) Group: *P* < 0.001 (F = 63.8) Time × Group: *P* < 0.001 (F = 8.2)
MDS‐UPDRS‐III (T_12M_)	19.5 ± 20.7^†^	20.7 ± 10.9	2.3 ± 1.8^§^, ^¶^	Time: *P* < 0.001 (F = 19.2) Group: *P* < 0.001 (F = 63.8) Time × Group: *P* < 0.001 (F = 8.2)

*Note*: Results are expressed as mean values±SD.

^†^ Posthoc *t*‐test between baseline and T_6M_ and T_12M_ in PD‐DBS (*P* ≤ 0.010).

^‡^ Posthoc *t*‐test between PD‐DBS and PD‐CON (*P* ≤ 0.011).

^§^ Posthoc *t*‐test between PD‐DBS and H‐CON (*P* < 0.001).

^¶^ Posthoc *t*‐test between PD‐CON and H‐CON (*P* ≤ 0.001).

Abbreviations: H‐CON, healthy control subjects; MDS‐UPDRS, Movement Disorders Society Unified Parkinson's Disease Rating Scale; PD‐CON, PD patients under best medical treatment; PD‐DBS, patients with STN DBS; T_0_, baseline measurement; T_6M_, after 6 months of stimulation; T_12M_, after 12 months of stimulation.

### Effect of STN DBS on Sleep‐Related Scales

#### PDSS

**FIG. 1:**
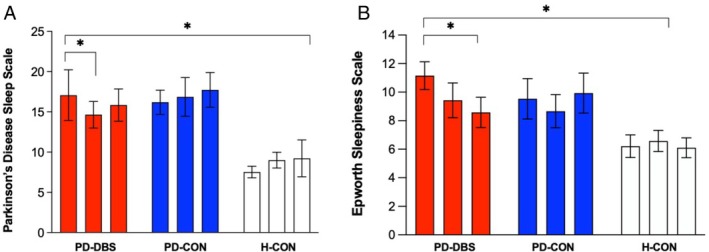
Changes in Parkinson's disease Sleep Scale (A) and Epworth Sleepiness Scale (B). Mean change in total scores as a comparison between groups and time points: baseline (T_0_, first bar per group), after six months (T_6M_; second bar per group), and after 12 months (T_12M_; third bar per group). PD‐DBS, patients with STN DBS (red bars); PD‐CON, PD patients under best medical treatment (blue bars); H‐CON, healthy control subjects (white bars). Values are shown as mean values ± SEM.

The PDSS score changed over time (*P* = 0.010) with differences between groups (time × group interaction: *P* = 0.031). PDSS scores decreased by 14% six months post‐surgery in PD‐DBS (*P* = 0.050; Fig. 1A), whereas it remained stable over time in both control groups (*P* ≤ 0.105). This improvement was positively associated with the activation of the motor part of the STN (r = 0.582, *P* = 0.037). The effect on PDSS was no longer present 12 months post‐surgery (*P* = 0.993).

#### ESS

Daytime sleepiness differed between groups with the lowest values in H‐CON (*P* = 0.006; F = 5.85). There was a time × group interaction (*P* < 0.001) with a reduction by 23% in the ESS score 12 months post‐surgery in PD‐DBS (*P* = 0.006; Fig. 1B), whereas both control groups remained stable over time (*P* ≥ 0.142). The decrease in ESS scores after 12 months of stimulation correlated with the activation of the limbic subdivision of the STN (r = 0.737, *P* = 0.006).

**FIG. 2:**
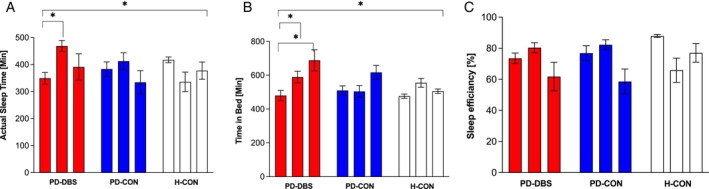
Changes in total actual sleep time (A) and total time in bed. Mean change in actual sleep time and time spent in bed as a comparison between groups and time points: baseline (T_0_, first bar per group), after six months (T_6M_; second bar per group), and after 12 months (T_12M_; third bar per group). PD‐DBS, patients with STN DBS (red bars); PD‐CON, PD patients under best medical treatment (blue bars); H‐CON, healthy control subjects (white bars). Values are shown as mean values ± SEM.

Total actual sleep time was not different between groups (p = 0.604), and did not change over time (time × group interaction; *P* = 0.215). For PD‐DBS, total actual sleep time increased from initially 5.82 hours up to 7.81 h after six months (*P* = 0.001; d = −1.49; Fig. 2A), which dropped again after further six months of stimulation (*P* = 0.995).

Time in bed changed over time (*P* = 0.001; F = 7.92), and the extent of change depended on the group (time × group interaction; *P* = 0.016; Fig. 2B). In PD‐DBS, the time in bed continuously increased after surgery from initially 8.00 h up to 9.82 h after six months and up to 11.47 h after 12 months. Posthoc test revealed a difference after 12 months post‐surgery (*P* = 0.006) with a positive correlation with the activation of the motor part of the right STN (r = 0.941, *P* = 0.005). In both control groups, time in bed did not change over time (all *P* ≥ 0.123).

Sleep efficiency changed over time (*P* = 0.034; F = 3.62), with no differences between the groups (*P* = 0.933), and the extent of change was not depending on group (time × group interaction; *P* = 0.099). In PD‐DBS, sleep efficiency increased after surgery from initially 73.6 ± 11.1% up to 80.4 ± 10.1% after six months of stimulation (*P* = 0.041; d = −0.66), but pointed to 61.8 ± 27.6 after 12 months (*P* = 0.173). The change in sleep efficiency after 6 months of stimulation correlated with the activation of the motor subdivision of the STN (r = −0.706, *P* = 0.033).

### Effect of STN DBS on Mood Changes

The BDI2 score changed over time (*P* = 0.038) with differences between groups (*P* < 0.001) as well as a time × group interaction (*P* = 0.034). The change in BDI2 scores were associated with the activation of the limbic part of the STN (r = −0.583, *P* = 0.017) after 6 months of stimulation. There was no correlation of BDI2 scores and the reduction in daily physical activity in PD‐DBS (r = 0.105, *P* = 0.555).

### Effect of STN DBS on Clock Gene Expression

**FIG. 3:**
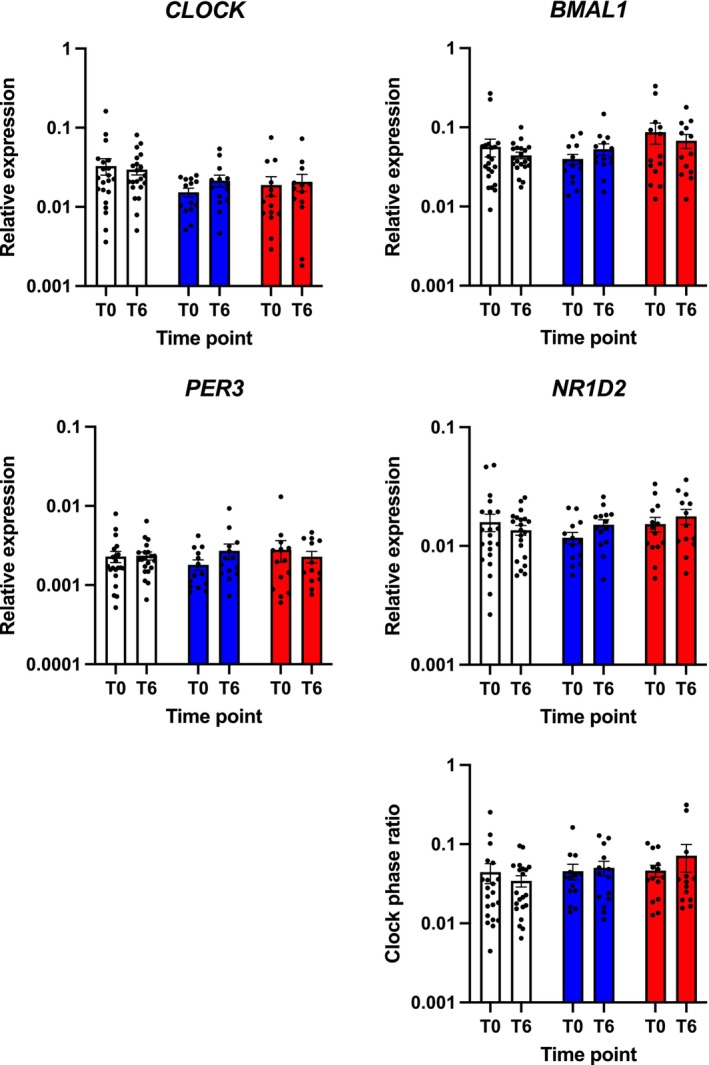
STN DBS does not significantly alter blood circadian clock gene expression. (A–D) Relative expression of clock genes *CLOCK* (A), *BMAL1* (B), *PER3* (C), and *NR1D2* (D) in whole‐blood at baseline (T0) and after six months (T6) determined by qPCR. (E) Positive components of the molecular circadian clockwork CLOCK/BMAL1 are expressed in anti‐phase to negative components PER3 and NR1D2. (F) Clock phase ratio (for details see text). PD‐DBS, patients with STN DBS (red bars); PD‐CON, PD patients under best medical treatment (blue bars); H‐CON, healthy control subjects (white bars). Values are shown as mean values ± SEM.

Circadian rhythmicity analysis showed neither an effect of DBS nor an effect of time, nor no difference at baseline in PD‐CON, on overall rhythmicity in all measured clock genes and in clock phase ratio (all *P* ≥ 0.323; Fig. 3A–F).

## Discussion

Sleep disorders are very common in PD and include disturbances of the sleep–wake‐cycle, excessive daytime sleepiness, and alterations in circadian rhythms.^1^, ^2^, ^3^, ^4^ The causes are multifactorial and comprise the re‐emergence of motor symptoms at night, side effects of dopaminergic medication, or nocturia.^3^ In the present study, the sleep–wake cycle was partly improved after STN DBS surgery, and this improvement was related to the position of the electrode within the STN. Since the expression of clock genes in blood was not changed, other factors possibly cause STN DBS‐related improvement in sleep.

### The Effect of Treatment on Sleep

Following STN DBS, most patients experience a reduction in their LED requirements. DBS itself leads to improved motor fluctuations, tremors, and reduced dyskinesias, which may positively influence sleep quality by reducing nocturnal symptoms and enhancing overall sleep continuity. Conversely, in some cases, patients may require adjustments to their medication regimen post‐surgery to address re‐emerging motor symptoms, potentially impacting sleep patterns.^12^ Here, we showed that STN DBS positively influenced several sleep parameters, mainly by reducing daytime sleepiness, which correlated strongly with activation of the limbic subdivision of the STN. Improvements in time in bed, sleep efficiency and PDSS at 6 months did not sustain at 12 months, probably explained by multiple factors, stemming not only from motor symptoms but also from non‐motor issues such as nocturia and neuropsychiatric disturbances. Also, dopaminergic medication adjustments after DBS could influence sleep–wake patterns. Additionally, long‐term habituation to stimulation or the natural progression of PD‐related non‐motor symptoms may attenuate initial sleep benefits. Finally, the precision of electrode placement and variability in the activation of specific STN subregions might influence the modulation of adjacent structures involved in sleep regulation.

Reduced daytime activity could reflect changes in physical activity levels associated with DBS, and other factors such as worsening apathy, mood disturbances, or the reduction in LED observed in our cohort may also play a role. We could show that changes in BDI‐2 scores were associated with stimulation of the limbic proportion of the STN. Factors such as persistent nocturnal symptoms (eg, nocturia) or the natural progression of PD may have contributed to the lack of sustained improvements in sleep measures.

It is reasonable to expect that alterations in dopaminergic medication after STN DBS might lead to changes in circadian processes due to essential dopamine functions in sleep regulation. DBS allows for a reduction in dopaminergic medication, with LEDD decreasing by 42% at 6 months in the PD‐DBS group and remaining stable thereafter. This reduction likely contributed to improving ESS by reducing medication‐induced daytime sleepiness as excessive dopaminergic stimulation can exacerbate daytime sleepiness through its effects on circadian and neurotransmitter systems. By mitigating medication‐induced fluctuations, DBS may furthermore promote a more stable sleep–wake cycle. However, the lack of sustained improvements in other sleep measures at 12 months suggests that factors beyond dopaminergic modulation such as persistent nocturnal symptoms, the natural progression of PD or long‐term habituation to DBS impact sleep.

Dopaminergic treatments often cause drug‐induced sleep disturbances, including insomnia, vivid dreams, and sudden onset of sleep cycle.^3^ The administration of levodopa and dopamine agonists affects sleep patterns in patients with PD, with fluctuations in dopamine levels throughout the day potentially disrupting normal sleep architecture during the night. In addition, dopamine directly affects circadian rhythms through receptors in the suprachiasmatic nucleus (SCN) and modulates the expression of clock genes.^3^, ^5^

In STN DBS‐treated patients, the exact postoperative electrode location of the active contact and the modulation of the electrical field directly influence adjacent structures that are involved in sleeping behavior and energy balance by excitation of axons surrounding the electrode in combination with increased output from stimulated nuclei.^23^, ^24^, ^25^, ^26^ An estimation of the spread of the current has been captured of approximately a 2–4 mm radius around the active electrodes.^27^, ^28^, ^29^ Moreover, given the functional and structural complexity of the basal ganglia circuitries,^30^ the current diffusion plays a role in the different basal ganglia circuitries, causing motor improvements but, on the other hand, also non‐motor side effects.^25^

Our findings indicate that electrode placement within the STN influences changes in specific sleep parameters. In contrast, enhancements in time in bed and sleep efficiency were associated with activation of motor subregions confirming that electrode position within the STN can differentially affect sleep outcomes.^31^, ^32^ Despite these differences, converging evidence underscores that distinct STN subregions contribute uniquely to specific sleep domains. The limbic STN appears to dominate in regulating wakefulness and daytime alertness, as reflected in our ESS results, whereas the motor and dorsal subregions may influence nocturnal sleep parameters.

The activation of the limbic subdivision of the STN was associated with a substantial reduction in excessive daytime sleepiness in our study. Through the connections of the STN to the ventral tegmental area and ventral pallidum, as important parts of the sleep regulatory circuitry, stimulation of the STN may thus increase dopaminergic conveyance in the striatum and enhance sleep.^33^, ^34^ In addition to improvement in PDSS total score it has been reported that overall sleep quality as well as specific sleep parameters improved following DBS.^12^ However, since only a few studies investigated the long‐term effects of STN DBS on autonomic function^3^, ^35^ and sleep,^36^, ^37^ no clear explanation for the effects of STN DBS on sleep can be drawn.

### Parkinson's Disease and Circadian Rhythms

PD patients show altered *BMAL1* and Neuronal PAS Domain Protein 2 (*NPAS2*) expression,^4^ also, altered melatonin secretion patterns and elevated cortisol levels also altered melatonin secretion patterns, and elevated cortisol levels that are linked to sleepiness, sleep architecture changes, and mood disturbances.^3^, ^38^, ^39^

Circadian rhythms, which control nearly all physiological systems, are orchestrated by a central pacemaker in the SCN and peripheral oscillators.^3^, ^38^ Disruption of circadian rhythms has been linked to a variety of health problems, such as metabolic and immune dysregulation, cognitive impairment, psychiatric and mood disorders.^40^ In PD, alterations in the circadian system contribute to both motor and non‐motor symptoms. Motor disturbances include increased bedtime activity and decreased daytime activity, and reduced evening motor response to levodopa,^4^ suggesting disrupted circadian regulation of dopaminergic systems. Non‐motor symptoms in PD include cardiovascular rhythm disturbances, and fluctuations in visual performance related to changes in retinal dopamine.^3^ Furthermore, single nucleotide polymorphisms in clock genes have been associated with PD incidence^5^ in *CLOCK, BMAL1, PER1, PER2, CRY1*, and *CRY2*. These genetic variations may contribute to PD pathogenesis by affecting the circadian modulation of mitochondrial bioenergetics, autophagy, and neuroendocrine functions and studies have demonstrated dysregulation of circadian gene expression.^3^

Several factors may explain the absence of changes in peripheral clock gene expression in the present study. The gene expression analysis in whole blood may not capture the localized effects of DBS on brain‐specific circadian regulators, such as the SCN or hypothalamus. Peripheral blood serves as a proxy, but it is subject to numerous confounding factors, including variability in immune cell populations and stress‐related hormonal influences, which may dilute subtle DBS‐induced changes. While DBS has been shown to modulate motor and limbic circuits, its impact on circadian‐regulating pathways may be indirect and not robust enough to induce measurable changes in peripheral gene expression.

### Metabolic Consequences of Sleep Alterations

Sleep disturbances are well‐known to impact metabolic health, contributing to obesity risk, impaired glucose tolerance, and hormonal dysregulation.^7^, ^8^, ^40^, ^41^ While this study did not directly assess obesity risk or related metabolic consequences, the observed changes in sleep duration and efficiency—particularly the transient improvements at 6 months—may contribute to altered energy balance in DBS‐treated patients and previous studies have demonstrated weight gain as a frequent consequence of DBS,^42^ potentially linked to motor improvements, reduced physical activity, or altered energy metabolism.

### Strength and Limitations

The negative results in linking changes in sleep and clock gene expression after STN DBS might be due to different reasons. First, minor alterations in gene expression may have escaped the assessment in peripheral blood.^43^ Since blood samples were taken only in the morning, the morning‐evening ratio could not be calculated, so more detailed information on diurnal gene expression patterns was unavailable. Moreover, this study represents a secondary, post‐hoc analysis of existing data. Despite providing power calculations, we acknowledge that the study may still have been underpowered to detect more subtle differences, particularly in clock gene expression and specific sleep parameters. With wrist actigraphy, we were able to assess sleep duration and quality under free‐living conditions, but not sleep architecture because this requires polysomnography. This should be encountered for future studies to objectify the effect of STN DBS on sleep stages as previously reported.^44^ Furthermore, the relatively small sample size of subjects with STN DBS must also be considered while a strength of the present study is the inclusion of the two control groups.

## Conclusions and Clinical Implications

Our findings are in accordance with previous research and support the hypothesis that the positive effect of STN DBS in patients with PD on sleep–wake cycle may, at least partially, be determined by a regional impact of stimulation on adjacent structures that are involved in the central regulation of sleep and energy balance. Advances in personalized DBS, such as closed‐loop systems and better adaptation of the therapeutic field within the STN, promise to improve disease symptoms and minimize side effects.^45^, ^46^

## Author Roles

(1) Research project: A. Conception, B. Organization, C. Execution; (2) Statistical Analysis: A. Design, B. Execution, C. Review and Critique; (3) Manuscript: A. Writing of the first draft, B. Review and Critique.

J.S.: 1C, 2A, 2B, 3A

L.L.: 1C, 2A, 2B, 3B

X.C.S.: 1C, 2B, 3B

D.R.: 1B, 2C, 3B

O.H.: 1C, 2B, 3B

B.W.: 1A, 1B, 1C, 2A, 2B, 3B

N.B.: 1A, 1B, 1C, 2A, 2B, 3B

## Disclosures

**Ethical Compliance Statement:** The study was approved by the local ethics committee of the University of Lübeck (AZ17‐198) and conducted according to the ethical standards of the Declaration of Helsinki. Before participation, informed written consent was obtained from all participants. We confirm that we have read the Journal's position on issues involved in ethical publication and affirm that this work is consistent with those guidelines.

**Funding Sources and Conflicts of Interest:** This publication was supported by a grant of the German Research Foundation to the Graduiertenkolleg 1957 “Adipocyte‐Brain Crosstalk,” University of Lübeck. The authors declare that there are no conflicts of interest relevant to this work. NB received honaria from Abbott, Abbvie, Biogen, Biomarin, Bridgebio, Centogene, Esteve, Ipsen, Merz, Teva, Zambon.

**Financial Disclosures for the Previous 12 Months:** NB received honaria from Abbvie, Merz and Teva.

## Supporting information


**Supplementary Table S1.** Stimulation settings for all patients individually described by contacts, voltage, pulse width, frequency, impedance, and current.

**Supplementary Figure S1.** Group‐level electrode localization. (A) 3D reconstruction showing electrode locations in both hemispheres. (B–D) Representative 2D views of the right hemisphere in axial (B), sagittal (C), and coronal (D) orientations. The subthalamic nucleus (STN) is shown in orange, and the red nucleus in red.

## Data Availability

The data that support the findings of this study are available on request from the corresponding author. The data are not publicly available due to privacy or ethical restrictions.
