# Supplementary material for: The Impact of Deep Brain Stimulation of the Subthalamic Nucleus on Sleep–Wake Function and Circadian Rhythms in Patients with Parkinson's Disease
Source: Mov Disord Clin Pract. 2025 Jun 4;12(11):1801–10. doi: 10.1002/mdc3.70160 (PMC12625183; doi:10.1002/mdc3.70160)
Supplement: Supplementary file 1 — Supplementary Table S1. Stimulation settings for all patients individually described by contacts, voltage, pulse width, frequency, impedance, and current. [file MDC3-12-1801-s002.docx]

| Code | Parameters of chronic DBS after 6 months of stimulation | | | | | | | | | | | | Parameters of chronic DBS after 12months of stimulation | | | | | | | | | | |
| --- | --- | --- | --- | --- | --- | --- | --- | --- | --- | --- | --- | --- | --- | --- | --- | --- | --- | --- | --- | --- | --- | --- | --- |
|  | Contacts | | Amplitude (V) | | Pulse width (µs) | | Hz | | Impedance (Ω) | | Current (mA) | | Contacts | | Amplitude (V) | | Pulse width (µs) | | Hz | Impedance (Ω) | | Current (mA) | |
|  | L | R | L | R | L | R |  | L | | R | L | R | L | R | L | R | L | R |  | L | R | L | R |
|  |  | | | | | | | | | | | |  | | | | | | | | | | |
| Patient 1 | 0- C+ | 10- C+ | 2.1 | 2.9 | 120 | 60 | 150 | 1338 | | 1144 | 1.6 | 2.5 | 0-1+ | 9- C+ | 2.1 | 3.1 | 120 | 160 | 180 | 1340 | 1112 | 1.6 | 2.8 |
| Patient 2 | 2- 3+ | 10- C+ | 1.7 | 2.4 | 60 | 60 | 130 | 2784 | | 1346 | 0.6 | 1.8 | 2- C+ | 9- C+ | 1.4 | 2.4 | 60 | 60 | 130 | 1027 | 985 | 0.9 | 2.2 |
| Patient 3 | 2- 3+ | 9- 10+ | 3.3 | 3.8 | 60 | 90 | 130 | 1367 | | 1396 | 2.5 | 2.8 | 2- C+ | 9-C+ | 2.6 | 2.7 | 60 | 60 | 130 | 2272 | 2344 | 1.1 | 1.2 |
| Patient 4 | 1- 2+ | 9- 10+ | 1.3 | 2.0 | 60 | 60 | 130 | 3201 | | 2412 | 0.4 | 0.8 | 1-2+ | 9-10+ | 1.7 | 2.4 | 60 | 60 | 130 | 1828 | 1626 | 0.9 | 1.5 |
| Patient 5 | 1-C+ | 9-C+ | 1.3 | 1.5 | 60 | 60 | 130 | 1247 | | 1144 | 1.1 | 1.3 | 1-C+ | 9-C+ | 1.3 | 1.8 | 60 | 60 | 130 | 1299 | 1155 | 1.0 | 1.6 |
| Patient 6 | 1-2+ | 9- 11+ | 2.5 | 2.8 | 60 | 60 | 180 | 2272 | | 2344 | 1.1 | 1.2 | 1-2+ | 9-11+ | 2.9 | 3.2 | 60 | 60 | 180 | 2257 | 2909 | 1.3 | 1.1 |
| Patient 7 | 1+2- | 9- 10+ | 3.5 | 2.4 | 60 | 60 | 130 | 1960 | | 2502 | 1.8 | 1.0 | 1+2- | 9-10+ | 4.3 | 3.1 | 60 | 60 | 130 | 1985 | 2880 | 2.2 | 1.1 |
| Patient 8 | 0-1+ | 8+9- | 2.9 | 3.5 | 90 | 90 | 130 | 1969 | | 2492 | 1.5 | 2.4 | 0-1+ | 8+9- | 3.0 | 3.8 | 90 | 90 | 130 | 2028 | 1801 | 1.5 | 2.4 |
| Patient 9 | / | 10-C+ | / | 4.5 | / | 60 | 130 | / | | 1087 | / | 4.1 | / | 10- C+ | / | 4.6 | / | 150 | 130 | / | 1066 | / | 4.3 |
| Patient 10 | 1-C+ | 9-C+ | 2.2 | 2.5 | 90 | 90 | 130 | 1657 | | 1397 | 1.3 | 1.8 | 1-C+ | 9-C+ | 3.0 | 2.5 | 60 | 90 | 130 | 1405 | 1366 | 2.2 | 1.8 |
| Patient 11 | 1-2+ | 9- C+ | 3.9 | 3.1 | 60 | 60 | 180 | 777 | | 1002 | 5.0 | 3.1 | 0-1+ | 9-C+ | 4.2 | 3.6 | 60 | 60 | 180 | 750 | 840 | 5.6 | 4.3 |
| Patient 12 | 2+1- | 10- C+ | 1.9 | 2.7 | 60 | 60 | 160 | 528 | | 914 | 3.5 | 2.9 | 1+2- | 10- C+ | 2.0 | 2.8 | 60 | 60 | 130 | 570 | 867 | 3.2 | 3.1 |
| Patient 13 | 2-C+ | 10-C+ | 2.2 | 2.7 | 60 | 60 | 130 | 1121 | | 1248 | 2.0 | 2.2 | 2-C+ | 10- C+ | 2.2 | 2.7 | 60 | 60 | 130 | 1121 | 1248 | 2.2 | 2.5 |
| Patient 14 | 0-1+ | 9-10+ | 3.7 | 4.1 | 90 | 90 | 130 | 2056 | | 1280 | 1.8 | 3.2 | 0-C+ | 10- C+ | 2.0 | 2.9 | 90 | 90 | 130 | 1598 | 988 | 1.3 | 2.9 |

Supplementary Table 1. Stimulation settings for all patients individually described by contacts, voltage, pulse width, frequency, impedance, and current

Notes. Stimulation settings for all patients individually described by contacts, voltage, pulse width, frequency, impedance, and current for chronic DBS.
