# Supplementary figures and images for: The Impact of Deep Brain Stimulation of the Subthalamic Nucleus on Sleep–Wake Function and Circadian Rhythms in Patients with Parkinson's Disease
Source: Mov Disord Clin Pract. 2025 Jun 4;12(11):1801–10. doi: 10.1002/mdc3.70160 (PMC12625183; doi:10.1002/mdc3.70160)

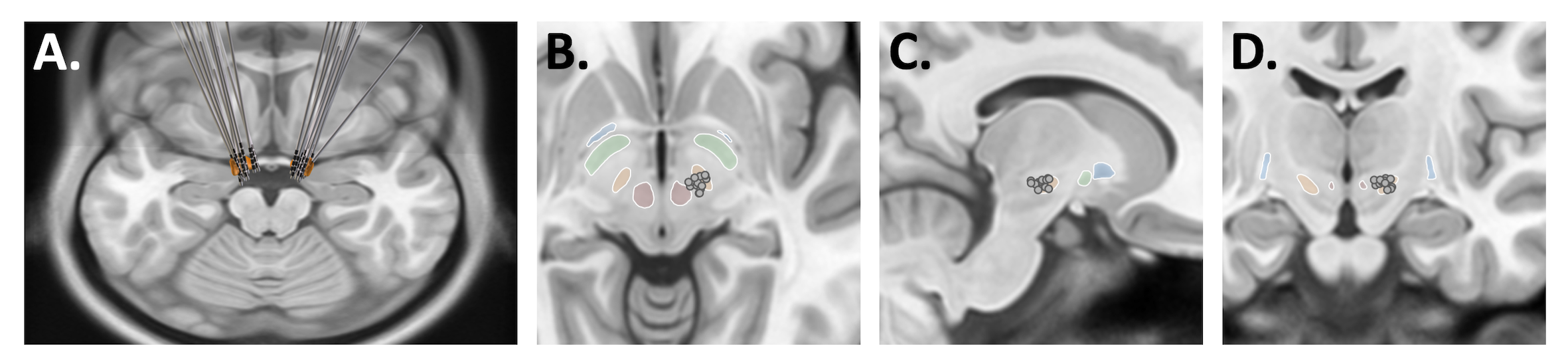

Supplement: Supplementary file 2 — Supplementary Figure S1. Group‐level electrode localization. (A) 3D reconstruction showing electrode locations in both hemispheres. (B–D) Representative 2D views of the right hemisphere in axial (B), sagittal (C), and coronal (D) orientations. The subthalamic nucleus (STN) is shown in orange, and the red nucleus in red. [file MDC3-12-1801-s001.tiff]
